# SINE RNA Induces Severe Developmental Defects in *Arabidopsis thaliana* and Interacts with HYL1 (DRB1), a Key Member of the DCL1 Complex

**DOI:** 10.1371/journal.pgen.1000096

**Published:** 2008-06-13

**Authors:** Marie-Noëlle Pouch-Pélissier, Thierry Pélissier, Taline Elmayan, Hervé Vaucheret, Drasko Boko, Michael F. Jantsch, Jean-Marc Deragon

**Affiliations:** 1 Université de Perpignan Via Domitia, CNRS UMR5096 LGDP, Perpignan, France; 2 INRA Laboratoire de Biologie Cellulaire, Versailles, France; 3 Department of Chromosome Biology, Max F. Perutz Laboratories, University of Vienna, Vienna, Austria; The University of North Carolina at Chapel Hill, United States of America

## Abstract

The proper temporal and spatial expression of genes during plant development is governed, in part, by the regulatory activities of various types of small RNAs produced by the different RNAi pathways. Here we report that transgenic *Arabidopsis* plants constitutively expressing the rapeseed SB1 SINE retroposon exhibit developmental defects resembling those observed in some RNAi mutants. We show that SB1 RNA interacts with HYL1 (DRB1), a double-stranded RNA-binding protein (dsRBP) that associates with the Dicer homologue DCL1 to produce microRNAs. RNase V1 protection assays mapped the binding site of HYL1 to a SB1 region that mimics the hairpin structure of microRNA precursors. We also show that HYL1, upon binding to RNA substrates, induces conformational changes that force single-stranded RNA regions to adopt a structured helix-like conformation. *Xenopus laevis* ADAR1, but not *Arabidopsis* DRB4, binds SB1 RNA in the same region as HYL1, suggesting that SINE RNAs bind only a subset of dsRBPs. Consistently, DCL4-DRB4-dependent miRNA accumulation was unchanged in SB1 transgenic *Arabidopsis*, whereas DCL1-HYL1-dependent miRNA and DCL1-HYL1-DCL4-DRB4-dependent tasiRNA accumulation was decreased. We propose that SINE RNA can modulate the activity of the RNAi pathways in plants and possibly in other eukaryotes.

## Author Summary

Short interspersed elements (SINEs) are transposable elements in eukaryotic genomes that mobilize through an RNA intermediate. Recently, mammalian SINE RNAs were shown to have roles as noncoding riboregulators in stress situations or in specific tissues. Mammalian SINE RNAs modulate the level of mRNAs and proteins by interacting with key proteins involved in gene transcription and translation. Here we show that constitutive production of a plant SINE RNA induces developmental defects in *Arabidopsis thaliana* and that this SINE RNA interacts with HYL1, a double-stranded RNA-binding protein required for the production of microRNA and trans-acting small interfering (tasi)RNA. We mapped the binding site of HYL1 to a SINE RNA region that mimics the hairpin structure of microRNA precursors. We also found that HYL1 induces conformational changes upon binding to RNA substrates. These data suggest that SINE RNAs modulate the activity of RNAi pathways in *Arabidopsis*.

## Introduction

Short Interspersed Elements (SINEs) are repetitive sequences, present in the genome of most eukaryotes and ancestrally derived from small functional RNAs (tRNAs, 7SL RNAs or 5S RNAs) [1]. SINEs can be transcribed by the RNA polymerase III (polIII) machinery [2]. They propagate in genomes following reverse transcription and integration due to their capacity to interact efficiently with the translation products of Long Interspersed Elements (LINEs) [3],[4], a family of active retrotransposons. SINEs copy number usually ranges from several hundred to several thousand in most eukaryotic species, except in mammals where tens of thousands up to millions of copies can be found [1].

Evaluating SINE impact on genome structure and gene expression has been the subject of numerous investigations in the past 20 years (reviewed in [1], [5]–[7]). Most of these studies have been conducted at the DNA level, by evaluating how SINE copies affect chromatin structure, DNA recombination, replication and transcription. The effect of SINE sequences in mRNAs and the corresponding impacts on splicing, editing, degradation and translation processes have also been evaluated. Recently, several SINE polIII-specific transcripts were shown to act as noncoding riboregulators of basic cellular processes, including transcription and translation, in stress situations or in specific tissues. In rodents, following heat shock, several members of the SINE B2 family are actively transcribed [8],[9]. The B2 SINE RNA was shown to interact with and inhibit the RNA polymerase II complex, leading to a general repression of gene transcription in this stress situation [8],[9]. The polIII-specific transcription of human Alu, rodent B1 and silkworm Bm1 SINEs can also be activated by several biotic and abiotic stresses [10]–[15]. Alu RNA was proposed to regulate translation either by modulating the activity of the Protein Kinase R (PKR), a double-stranded RNA binding protein (dsRBP) that down-regulates translation in stress situations [10], or by a PKR-independent process [16],[17]. Recently, human Alu RNA was also shown to act as a modular transacting repressor of mRNA transcription during heat shock [18]. The rodent BC1 and human BC200 SINE-related elements are transcribed specifically in neurons where they regulate translation. BC1 and BC200 RNAs could potentially act as guides for the RNA-binding FMRP protein and regulate the translation of a small subset of neuron mRNAs [19],[20] although this mode of action was recently contested [21]. These RNAs can also have a more general impact on neuron translation by trapping essential translation factors such as eIF4B and PABPs [22],[23]. These different examples reveal that certain SINE loci produce non-coding regulatory RNA molecules that act on basic cellular functions. In this respect, SINE RNAs are similar to other polIII-transcribed riboregulators such as the cellular 7SK RNA regulating transcription elongation [24] and the viral VA1 and EBER1 RNAs regulating translation by interacting with PKR [25].

The *Arabidopsis thaliana* genome possesses six different SINE families representing a total of 334 repeated copies [26],[27]. In a previous study, we introduced a single copy of a *Brassica napus* SINE founder locus (SB1) under the control of its natural promoter in *Arabidopsis* and followed SINE RNA production and maturation in two independent transgenic lines [28]. Here we present evidence that the constitutive production of SINE RNA in these *Arabidopsis* lines can induce severe developmental defects. The SINE-induced phenotypes are similar to several RNAi mutant phenotypes. We show that SINE RNAs interact with a subset of highly divergent dsRBPs and affect the production of different families of small RNAs and the accumulation of their corresponding mRNA targets. Our results suggest that SINE RNAs influence the activity of a subset of dsRBPs and consequently, influence a variety of basic cellular processes including RNAi.

## Results

### SINE RNA Induces Developmental Defects in *Arabidopsis*

Fourteen *Arabidopsis thaliana* transgenic lines transformed with the founder SB1.7 (na7) locus from *Brassica napus* [2],[29],[30] were generated from two independent transformation experiments. We observed that most T2 individuals from nine of the fourteen transgenic lines displayed an apparent and similar developmental phenotype. To further characterize this phenotype, two transgenic lines (Col0-SB1.7(4) and Col0-SB1.7(18)), one for each independent transformation experiment, were selected. Both lines contained a single integration locus and were established at the homozygous state (data not shown).

**Figure 1:**
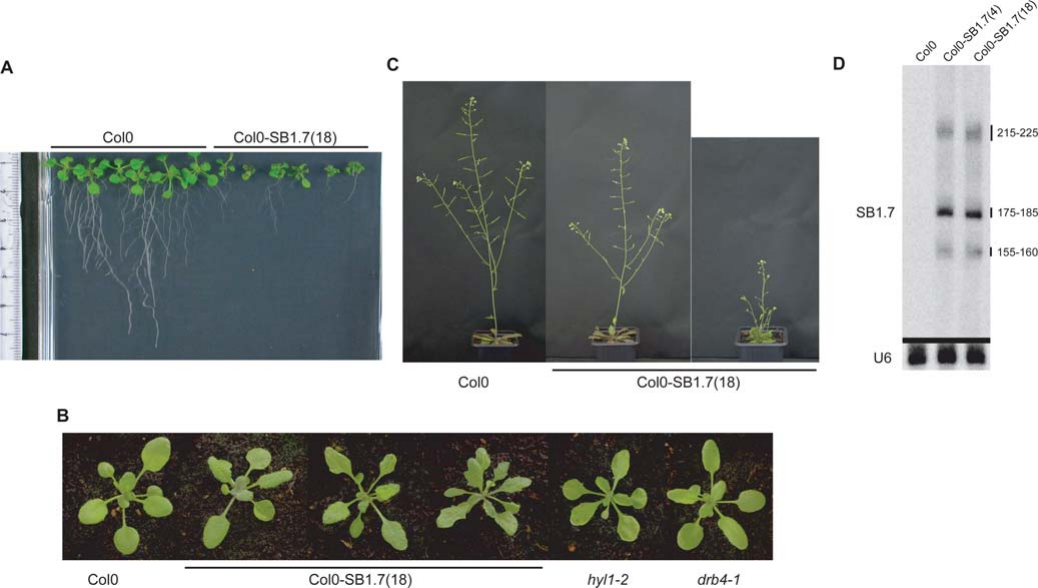
Description of the SINE-induced phenotype. Different individuals from the Col0-SB1.7(18) transgenic line producing SB1 RNA are compared to wild type (Col0) plants. A. Impact on root growth. Col0-SB1.7(18) individuals have much shorter roots compared to the wild type. Six different Col0-SB1.7(18) individuals representing the variability in this line are presented B. Comparison of 27 days seedlings from Col0, Col0-SB1.7(18) and *hyl1-2*, *drb4-1* RNAi mutant lines. Leaves from Col0-SB1.7(18) individuals are narrower, irregular in shape, and present a downward curvature. Three different Col0-SB1.7(18) individuals representing the variability in this line are presented. C. Col0-SB1.7(18) individuals have a general delayed growth, present shorter siliques and suffer from a partial loss of apical dominance. Two different Col0-SB1.7(18) individuals representing a mild and a severe case are presented. D. Typical pattern of SB1.7 SINE expression obtained by PAA gel hybridization after an 18h exposure. Three SB1.7-specific RNA species were detected, as expected from the post-transcriptional processing of the SINE primary transcript [28]. The sizes (in nucleotides) of the hybridizing SINE RNA species are shown. Following stripping of the probe, the membrane was re-hybridized with a U6-specific probe and exposed for 10 min.

The SB1.7 locus contains transcriptional cis-enhancer motifs that allow the SINE to partially escape transcriptional repression in its natural host [2]. SINE SB1 primary transcripts and maturation products were detected in the two transgenic lines by Northern hybridization followed by a 18 to 48 hour exposure time [28]. The level of SB1 RNAs in these lines is therefore much lower compared to other endogenous polymerase III products such as U6 RNA, which only require a few minutes of exposure after hybridization under identical conditions (see Figure 1D). The global severity of the developmental defects was variable between the two lines. Also, the penetrance of the phenotype was variable within each transgenic line, as plants with relatively mild to severe developmental defects were observed in each population (see Figure 1 for examples). Selfing plants with severe developmental defects gave progenies composed again of a mixture of plants with mild to severe developmental defects. The same result was observed when plants with mild defects were selfed, suggesting that the severity of the phenotype is somehow determined by a stochastic process during development.

In *Arabidopsis*, SB1 transcription is associated with delayed growth and flowering time, abortive siliques, partial sterility, reduction of leaf and root size, leaf serration associated with a downward curvature, and partial loss of apical dominance (Figure 1). Several of these defects resemble those observed in *hyl1* and *drb4* mutants, which are impaired in the two dsRBPs required for miRNA and trans-acting small interfering RNA (tasiRNA) pathways, respectively (see Figure 1B and 1C), suggesting that SB1 RNA could interact with RNA-binding proteins of the miRNA or tasiRNA pathways.

### SINE RNA Interacts with a Subset of Double-Stranded RNA Binding Proteins

To explain the observed similarity between SB1 expressing lines and RNAi mutants, we hypothesized that if SB1 RNA mimicked the structure of natural mi/tasiRNA substrates, it could interact with and titrate proteins involved in the biogenesis of these small RNAs (Figure 1). While SINEs derived from 7SL RNA (including mammalian Alu and B1) conserve the RNA folding of the ancestral molecule [31],[32], this is usually not the case for tRNA-derived SINEs like SB1 [33]. Indeed, using enzymatic and chemical probing approaches, we recently confirmed that SB1 RNA do not conserve the ancestral tRNA folding pattern but instead adopt a structure consisting of three stem-loops with bulges and mismatches [33]. This SB1 RNA secondary structure raises the possibility that it could interact with dsRBPs given that the recognition of dsRNA by dsRBPs generally does not involve sequence specificity and several structured RNAs forming stem-loops with bulges or mismatches were shown to bind efficiently to dsRBPs [34],[35]. The *Arabidopsis* genome has 19 dsRBPs, many of which are involved directly in RNAi [36]. These proteins include the four DICER-LIKE proteins (DCL1 to 4), the five dsRNA-BINDING PROTEINS (HYL1 and DRB2 to 5) and the HUA ENHANCER1 (HEN1) protein. Because the production of SINE RNA induces development defects that are similar to those of *hyl1-2* and *drb4-1* null mutants (Figure 1), we tested the capacity of SINE RNA to bind to HYL1 and DRB4.

**Figure 2:**
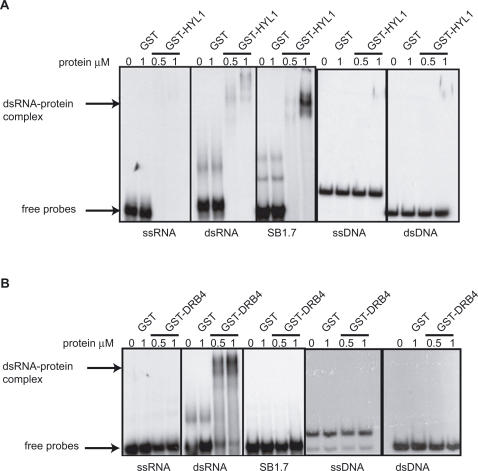
SINE RNA can bind to a subset of dsRBPs. A. Gel retardation experiments using GST, a recombinant GST-HYL1 fusion protein and [α-^32^P]-labeled single-stranded RNA, perfect double-stranded RNA, SB1.7 RNA, single-stranded DNA and double-stranded DNA B. Gel retardation experiments using GST, a recombinant GST-DRB4 fusion protein and [α-^32^P]-labeled single-stranded RNA, perfect double-stranded RNA, SB1.7 RNA, single-stranded DNA and double-stranded DNA. In both cases the amount of recombinant proteins used is indicated (in µM).

**Figure 3:**
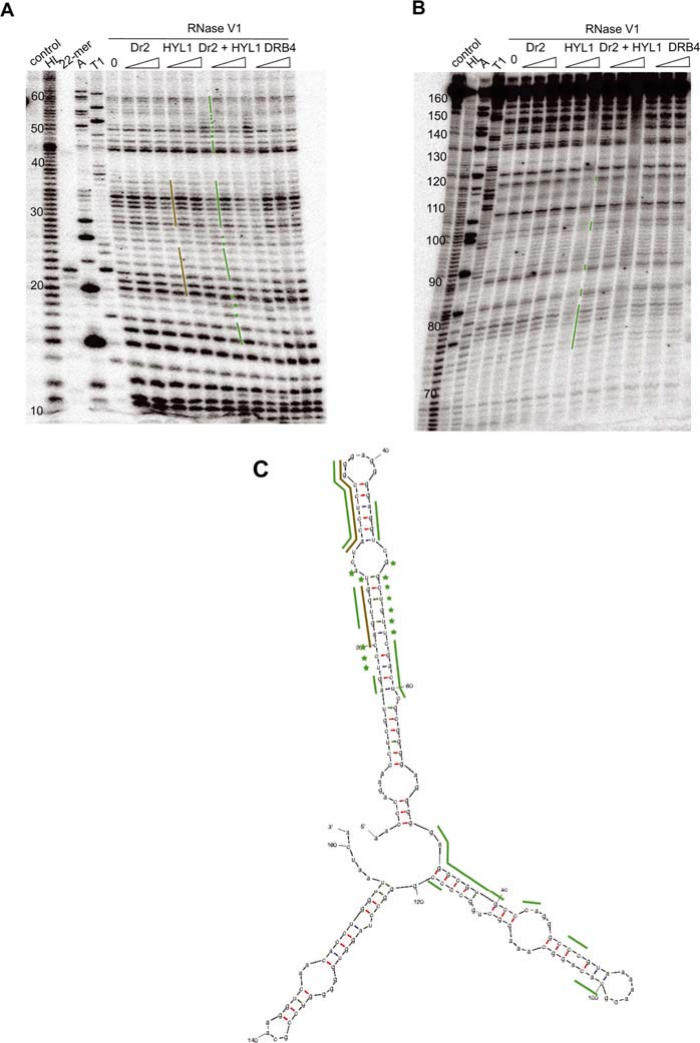
Protection from RNase V1 digestions of SINE RNA by two different dsRBPs. Prior to RNase V1 digestion, *in vitro* transcript of SB1 was subjected to protection by increasing concentrations of expressed dsRBPs: Dr2 (the second dsRBD of *Xenopus laevis* ADAR1), HYL1, combination of Dr2 and HYL1, or DRB4. Regions protected by Dr2 and HYL1 are marked alongside short run gel (A), long run gel (B) and predicted folding pattern (C) by brown and green bars, respectively. Three independent experiments gave similar results as the one presented. Nucleotides marked with asterisks seem to adopt more prominent helical structure upon protein binding and, therefore, become more prone to RNase V1 cleavage. DRB4 is showing no effect on RNase V1 cleavage, confirming its low *in vitro* binding affinity to SB1 RNA. Dr2 and HYL1, in this case, bind to and protect similar regions of RNA. HYL1 is, however, showing stronger binding affinity than Dr2. (HL) represents a partially hydrolyzed RNA ladder. Denaturating RNase A and T1 digests give the position of pyrimidine and G residues respectively. The control lane shows untreated RNA samples and the (0) lane represent RNase V1 digestion without recombinant proteins added. A labeled 23-mer oligoribonucleotide was also loaded on the gel to help in band size determination.

HYL1 is part of the DCL1 complex and is involved in processing miRNA primary transcripts (pri-miRNAs) and short precursors (pre-miRNAs) [37]–[40]. In gel retardation experiments, we observed that SB1 SINE RNA, but not DNA or single-stranded RNA fragments of a similar size, associate with a recombinant GST-HYL1 fusion protein (Figure 2A). Although a perfect RNA duplex also could bind HYL1, this association was less efficient compared to SB1 RNA, suggesting that HYL1 prefers dsRNA substrates containing unpaired nucleotide bulges and/or distal loops (Figure 2A). Using an RNase V1 protection assay, we defined more precisely the SB1 RNA binding sites of HYL1 (Figure 3). We observed that HYL1 binds mainly to the first and longest SB1 stem-loop, which corresponds to the region of SB1 RNA that adopts a fold similar to pre-miRNAs. Indeed, the protected region includes an RNA duplex containing mismatches and extends into the single-stranded terminal loop region (Figure 3C). A structurally similar, although weaker HYL1 binding site also is present on the second stem-loop of SB1 RNA (Figure 3). We also tested the capacity of DRB4, a dsRBP involved in the production of tasiRNAs [37] to bind SB1 RNA. In this case, the GST-DRB4 fusion protein did not bind significantly to SINE RNA in our *in vitro* assay, although it did efficiently bind to a perfect RNA duplex, which likely resembles the structure of tasiRNA templates (Figure 2B). This result suggests that SINE RNAs interact with dsRBPs specifically adapted to bind imperfect double-stranded RNA rather than those that bind perfect RNA duplexes.

Recently, double-stranded RNA binding domains from two *Xenopus laevis* proteins, xlADAR1 and xlRPBA, were shown to bind efficiently to short stem-loop RNA structures containing bulges and mismatches [41]. For xlADAR1, this result is consistent with the observation that ADARs can bind and modify miRNA precursors *in vivo* [42]. Because most tRNA-derived eukaryotic SINEs can adopt an RNA structure similar to SB1 RNA, [33] it is possible that many SINE RNAs interact with dsRBPs. We performed binding experiments with SB1 RNA and the second double-stranded RNA binding domain of xlADAR1 (called Dr2) and mapped the RNA binding sites. We observed that Dr2 bound SB1 RNA in the same region as HYL1 (Figure 3), suggesting that SINE RNAs have the potential to interact with a subset of dsRBPs across eukaryotic species, including the ones involved in miRNA production. DRB4 had no impact on RNase V1 cleavage pattern, confirming its inability to bind SB1. Also, no obvious enhancing (synergetic) effect was observed when Dr2 and HYL1 were used in the same binding experiment (Figure 3).

### HYL1 Induces Conformational Changes upon Binding to RNA

**Figure 4:**
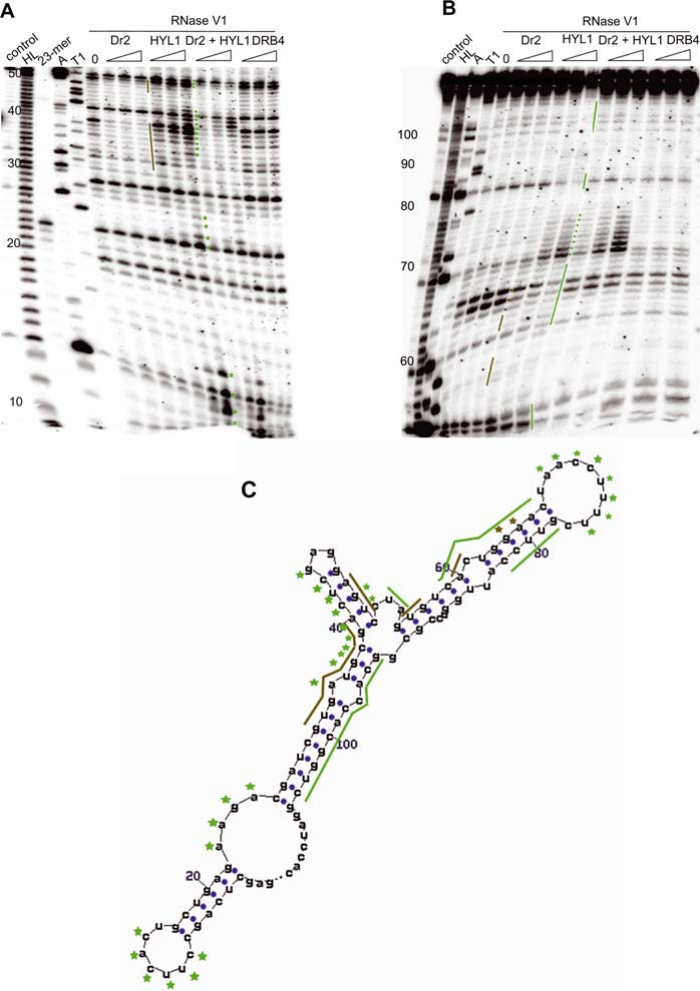
Protection from RNase V1 digestion of SELEX clone 11Dr2(7) by different dsRBPs. Prior to RNase V1 digestion, *in vitro* transcript of SELEX clone 11Dr2(7) was subjected to protection by increasing concentrations of expressed dsRBPs: Dr2, HYL1, combination of Dr2 and HYL1, or DRB4. Regions protected by Dr2 and HYL1 are marked alongside short run gel (A), long run gel (B) and predicted folding pattern (C) by brown and green bars, respectively. Three independent experiments gave similar results as the one presented. Nucleotides marked with asterisks seem to adopt more prominent helical structure upon protein binding and, therefore, become more prone to RNase V1 cleavage. DRB4 is showing no effect on RNase V1 cleavage of given RNA, confirming its low binding affinity for imperfect RNA duplexes. (HL) represents a partially hydrolyzed RNA ladder. Denaturating RNase A and T1 digests give the position of pyrimidine and G residues respectively. The control lane shows untreated RNA samples and the (0) lane represent RNase V1 digestion without recombinant proteins added. A labeled 23-mer oligoribonucleotide was also loaded on the gel to help in band size determination.

The binding of HYL1 appears to increase the RNase V1 sensitivity of certain regions of the SB1 RNA (indicated by asterisks on Figure 3A). The RNase V1 activity is sensitive to RNA conformation and, although sensitivity does not always imply hydrogen bonding of the bases in a canonical double stranded helix, it does require a structured, helix-like conformation [43]. As such, the increased RNase V1 sensitivity following HYL1 binding suggests that HYL1 is able to force some single-stranded RNA regions to adopt a more structured helix-like conformation, possibly by promoting non Watson-Crick base pairing. To test a chaperon-like activity for HYL1 and to explore its generality, we performed binding experiments using the SELEX clone 11Dr2(7), a short imperfect double-stranded RNA known to bind the Dr2 motif [41]. Following RNase V1 digestion, we confirmed the binding of Dr2 to 11Dr2(7) (Figure 4). We observed that HYL1 is able to bind strongly to 11Dr2(7) and generate regions protected from RNase V1 activity (represented by green lines on Figure 4B) and regions with increased sensitivity to RNase V1 (represented by asterisks on Figure 4A and B). Again DRB4 was unable to bind 11Dr2(7) and no synergetic effect was observed when HYL1 and Dr2 were used together. Similar results were observed when the SB2 *Arabidopsis* SINE RNA was used as a substrate (see Figure S1). Our results suggest that, upon binding RNA, HYL1 has the general capacity to force single-stranded regions to adopt a more organized, helix-like configuration.

### Molecular Impact of SINE RNA on the miRNA and tasiRNA Pathways

**Figure 5:**
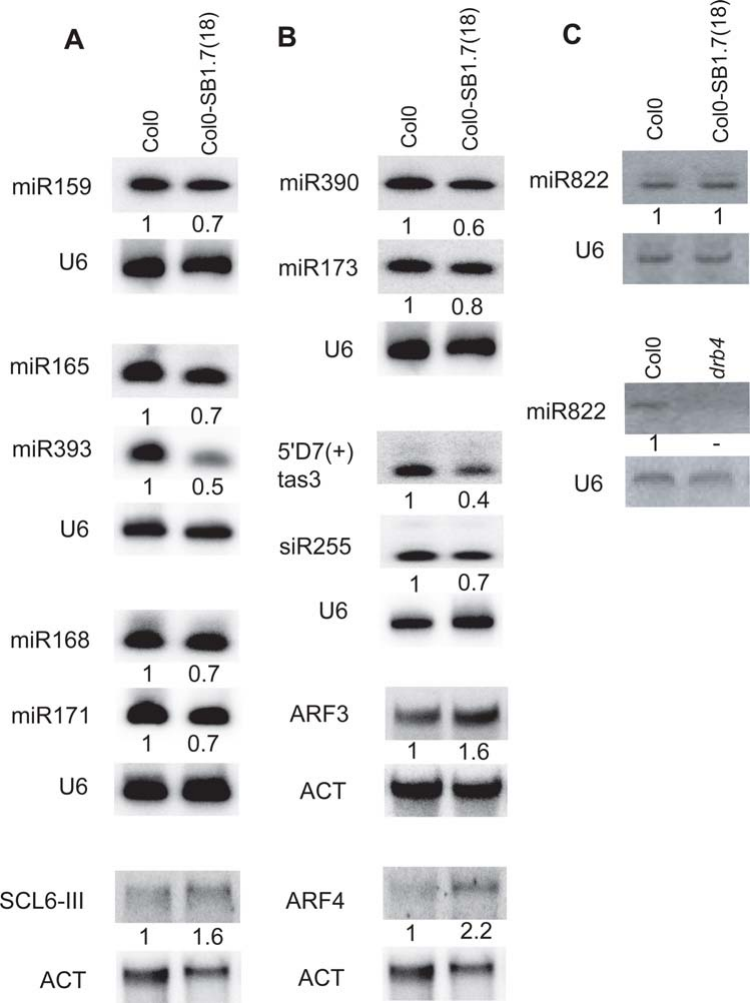
Molecular impact of SINE transcription on the different mi/tasiRNA pathways. A. Molecular impact on the miRNA pathway. Examples of five miRNAs that accumulate to lower levels in flowers from SINE expressing individuals (Col0-SB1.7(18)) compared to wild type (Col0). The correlative increase of the miR171-targeted SCL6-III mRNA is shown. B. Molecular impact on the tasiRNA pathway. The two miRNAs known to prime the synthesis of the tasiRNA precursors (miR173 for TAS1 and 2 and miR390 for TAS3) accumulate to a lower level in flowers from SINE expressing individuals (Col0-SB1.7(18)) compared to wild type (Col0). Consequently mature tasiRNA products (5′D7(+) TAS3, siR255 TAS1) are less abundant in SINE expressing individuals and messenger RNA targets of TAS3 (ARF3 and ARF4 mRNAs) are over-represented. C. The accumulation of the DCL4-DRB4-dependent miR822 [54] is unchanged in the Col0-SB1.7(18) transgenic line but miR822 is undetectable in the *drb4* mutant line. The relative proportion of miRNA, tasiRNA and mRNAs (the mean of at least three experiments) normalized using the U6 RNA or Actin mRNA signal is indicated. Similar results were obtained using the Col0-SB1.7(4) transgenic line.

To determine the molecular consequences of SB1 expression on the miRNA and tasiRNA pathways, we analyzed small RNA accumulation in our SB1 expressing lines. DCL1-HYL1-dependent miRNA accumulation was reduced in the two SB1 transgenic lines (Figure 5 and data not shown). Reduced miR171 accumulation coincided with increased accumulation of its target SCL6-III RNA (Figure 5A), suggesting that SB1 RNA could compete with miRNA precursors for HYL1 binding and thus reduce miRNA processing efficiency and miRNA-mediated regulation in planta. Consistent with the inability of SB1 RNA to bind DRB4 in gel retardation experiments (Figure 2), accumulation of DCL4-DRB4-dependent miRNA was unchanged in SB1 transgenic lines (see Figure 5C). The accumulation of tasiRNA also was reduced in SB1 transgenic lines, presumably because tasiRNA production primarily relies on the action of DCL1-HYL1-dependent miRNA miR173 and miR390 (Figure 5B). Indeed, reduced miR390 accumulation was consistent with reduced TAS3 tasiRNA levels and increased accumulation of TAS3 tasiRNA targets ARF3/ARF4 mRNAs (Figure 5B). No change in HYL1, DCL1 and HEN1 mRNA accumulation was detected in the Col0-SB1.7(18) SINE expressing line (see Figure S2) suggesting that the observed reduction in miRNA levels in this line does not result from repression of these miRNA pathway genes, and instead directly results from SINE RNA interaction with HYL1.

## Discussion

### SINE-Induced Developmental Defects

SB1 expressing lines display a diversity of phenotypes, suggesting that many important developmental transition steps are affected in these plants. The variable phenotypic penetrance within each line also suggests a stochastic effect of the RNA on these transition steps. Although we do not know the precise molecular mechanism(s) responsible for these phenotypes, our data raise the possibility that an interaction between SB1 SINE RNA and a subset of dsRBPs, some of which are involved in RNAi, could contribute to the developmental defects. Using a double-stranded RNA binding domain from *Xenopus laevis* ADAR1, we have shown that SB1 RNA is fit to bind highly divergent dsRBPs, and therefore many dsRBPs could be affected by SINE RNA expression. Not all *Arabidopsis* dsRBPs are known to be involved in RNAi. For example, FIERY2 is a dsRBP involved in transcriptional regulation [44], while two other dsRBPs of unknown function (At1g48650, at5g04895) contain a helicase domain. Therefore SB1 RNA potentially affects basic cellular processes other than RNAi, and this in turn could affect plant development.

Based on our *in vitro* studies, we propose that SB1 RNAs interact *in vivo* with HYL1, and consequently modify the steady-state level of several miRNAs and tasiRNAs. In this scenario, tasiRNA accumulation would be indirectly affected because tasiRNA biogenesis relies on miRNA-guided cleavage (miR173 targets TAS1 and TAS2 and miR390 targets TAS3) [45] (Figure 5B). DCL4-DRB4-dependent miRNA accumulation is unaffected in SB1 transgenic lines (see Figure 5C), consistent with the inability of SB1 RNA to bind DRB4 in gel retardation experiments. The SB1/HYL1 interaction and its molecular consequences on the miRNA pathway are unlikely to be solely responsible for the observed SB1-induced phenotype. Indeed, the global reduction of miRNA levels in SINE-expressing lines is generally moderate to low (from 0.5 to 0.8 of the initial amount) and this level of reduction does not always correlate with detectable increases in corresponding mRNA target levels (for examples see [46]). These relatively modest changes might be because several of these miRNAs derive from multigene families and thus potentially arise from several RNA precursors. In such cases, SINE RNAs would need to compete with several differently structured RNA precursors to limit effectively miRNA production. Also, *in vivo*, such competitions likely are influenced by the varying tissue/spatial distributions of different miRNAs:HYL1 complexes, which would probably effect the capacity of SINE RNA to modulate the production of a given miRNA in a given tissue. In conclusion, we propose that SB1 RNA compete for several dsRBPs, not only HYL1, and that these competitions likely accounts for the extent and unusual characteristics (such as the variable penetrance) of the SINE-induced phenotype.

### SINE RNA Can Bind HYL1 but not DRB4

We observed that, *in vitro*, HYL1 binds efficiently different imperfect double-stranded RNA molecules, including the rapeseed SB1 (Figures 2 and 3) and the *Arabidopsis* SB2 (Figure S1) SINE RNAs, while DRB4 only binds perfect RNA duplexes (Figures 2 and 3). These results are fully compatible with the known natural substrates of these two proteins. *In vivo*, HYL1 is known to interact with pri- and pre-miRNAs, which are organized as stem-loops containing mismatches and bulges (see the miRBase http://microrna.sanger.ac.uk/ for examples of pre-miRNA structures). On the other hand, DRB4 binds perfect linear RNA duplexes formed by the action of RDR6 on a single stranded primary transcript [36]. The fact that HYL1 does not play a major role in double-stranded RNA-induced posttranscriptional gene silencing (PTGS) [46] further suggests that, *in vivo*, HYL1 preferentially interacts with imperfect double-stranded pri- and pre-miRNAs and not perfect double-stranded PTGS precursors. Based on our RNase V1 mapping results, the basis of this selectivity could be the capacity of HYL1 to interact with single-stranded RNA regions (Figures 3 and 4). Indeed, the binding specificity of other eukaryotic dsRBPs, such as ADARs and Staufen, was shown to depend on their ability to interact with single-stranded RNA loops [47]. We therefore suggest that HYL1 has intrinsic RNA binding specificities distinct from DRB4, and that these specificities dictate different *in vivo* binding preferences.

### A Role for the Chaperoning-Like Activity of HYL1?

We also observed that upon binding RNA HYL1 has the general capacity to force single-stranded regions to adopt a more organized, helix-like configuration (Figures 3, 4 and Figure S1). *In vivo*, HYL1 mainly is involved in promoting processing steps from pri-miRNA to pre-miRNA in association with DCL1, another dsRBP [38],[39]. HYL1 also influences the cleavage positioning of DCL1 on the pre-miRNA to generate the mature miRNA [40]. Consequently, in the *hyl1-2* null mutant, pri-miRNAs accumulate and misplaced cleavages of pre-miRNAs were observed in some cases [38]–[40],[46]. However, HYL1 is not fully necessary for plant miRNA processing by DCL1 because the *hyl1-2* mutant retains some ability to accumulate wild type miRNAs, although the accumulation level is reduced. Also, this reduction is variable depending on the different miRNAs [46],[48]. HYL1 may therefore promote, to variable extents, the processing activity of DCL1. Based on our observations, one way HYL1 could do this is by inducing a conformational change in the RNA structure, forcing key single stranded regions to adopt organized, helix-like, configurations, including non Watson-Crick base pairing. This could in turn be important for promoting the cleavage activity of DCL1 on pri-miRNAs or for helping to precisely define the cleavage site on pre-miRNAs to generate mature products. It remains to be determined whether the chaperoning-like activity of HYL1 is important for miRNA production.

### Possible Evolutionary Consequence of the SINE RNA/dsRBP Interaction

We recently observed that related structural motifs are present in most SINE RNAs from mammals, fishes and plants, suggesting common selective constraints imposed at the SINE RNA structural level [33]. Using a double-stranded RNA binding domain from *Xenopus laevis* ADAR1, we have shown here that the plant SB1 RNA is fit to bind highly divergent dsRBPs. Therefore, the common trend of structural evolution observed for tRNA-related SINE could result in similar constraints imposed by a subset of dsRBPs across eukaryote species. If true, this predicts that SINE RNAs are under selective pressure to keep intact their capacity to interact with some dsRBPs. This would in turn forge the SINE RNA structure and impose, as observed [33], a common evolutionary history for most eukaryote tRNA-related SINEs. The reason why SINE RNA/dsRBP interaction would be under positive selective pressure is unclear, but precise and punctual expression of SINEs during a key development step or in a stress situation, could induce genetic and/or epigenetic variations and increase diversity and/or adaptability. It is interesting to note that SINE-specific expression in their natural host is highly regulated at the transcriptional and post-transcriptional levels by complex genetic and epigenetic processes (reviewed in [1],[10],[28]). SINEs are non-autonomous in their mobility and need the activation of an autonomous LINE partner to retrotranspose. Therefore, based on our results, we suggest that the main purpose of limiting SINE-specific transcription is not to prevent its mobility (the control of LINEs is sufficient to achieve this) but to preserve cell homeostasis by preventing SINE RNA to interact with a large subset of dsRPBs.

## Materials and Methods

### Plant Material

The construction of the SB1 expressing transgenic lines [28] and the *hyl1*, *drb4* mutant phenotypes [48],[49] were described previously. Plants were cultivated on soil in a greenhouse in standard conditions. For the study of root growth, plants were cultivated on germination medium (1 time MS salts; 10 g l^−1^ sucrose) plates at 21°C under a 12-h light/12-h dark regime.

### RNA Structure

RNA structures were predicted using RNA/DNA folding and hybridization software Mfold, version 2.3 [50]. For the SB1 and SB2 RNAs, the predicted structure was confirmed experimentally using chemical and enzymatic probing [33].

### Isolation of the HYL1 and DRB4 cDNAs and Purification of the Recombinant Proteins

cDNAs encoding HYL1 or DRB4 were amplified by PCR from an *Arabidopsis* cDNA library (Stratagene) using primers designed according to the *Arabidopsis* sequence database. All PCR amplifications were performed using 5′-primers with a terminal *Bam*HI restriction site in combination with a 3′-primer ending with a *Xho*I restriction site. After PCR amplification and *Bam*HI/*Xho*I digestion, the coding sequences of HYL1 or DRB4 were cloned into the pGEX-5X-1 expression vector (Pharmacia Biotech). In the resulting constructs named pGEX-HYL1 or pGEX-DRB4, HYL1 or DRB4 are fused to the C-terminal end of GST. Prior to expression in bacteria, sequencing was performed to verify the sequence of the cDNAs and the translational fusions. To express the HYL1 or DRB4 recombinant proteins, pGEX-HYL1 or pGEX-DRB4 were transformed into *Escherichia coli* BL21 (DE3) cells. A single colony of *E. coli* cells containing a recombinant pGEX plasmid was used to inoculate 50 ml of LB medium containing 100 µg/ml ampicillin. Cells were incubated overnight at 37°C with vigorous shaking. Cultures were diluted 1∶100 into fresh LB medium containing 50 µg/ml carbenicillin and grown at 37°C with shaking until the A_600_ reaches 0.7–1. Recombinant protein expression was then induced by addition of 0.1 mM isopropyl-ß-D-thiogalactopyranoside (IPTG) followed by an incubation of 4 to 5 hours at 37°C. Induced cells were harvested by centrifugation at 7700 *g* for 10 min at 4°C and pellets were frozen at −20°C overnight. Bacterial sonication and batch purification of the fusion proteins using Glutathione Sepharose 4B were performed according to the manufacturer's protocol (Pharmacia Biotech).

### Mobility Shift Assays

#### Nucleic Acid Preparation

SB1.7 was *in vitro* transcribed from linearized T7 promoter-containing vector using recombinant T7 polymerase and resulting transcripts were gel-purified. SB1.7 RNA was then dephosphorylated using shrimp alkaline phosphatase (Roche) according to the manufacturer's protocol and 5′-end labeled with T4 polynucleotide kinase (New England Biolabs) and [γ-^32^P] ATP (GE Healthcare). After labeling, SB1.7 RNA was purified using BD Chroma spin-30 columns (BD Biosciences Clontech). A pBluescript II SK vector (Stratagene) containing a short fragment (multiple cloning sites) between the T7 and T3 promoters was used to prepare substrates. The T3 transcript (approx. 110 nucleotides, prepared as described above for SB1.7 RNA) was used as the ssRNA substrate. To make the dsRNA substrate, T3 transcripts were first kinase-labeled, and then T7 transcripts were hybridized with labeled T3 transcripts to form dsRNA. A *Bss*HII fragment (173 base pairs) that included both phage promoters and the multiple cloning sites of the pBluescript II SK vector was gel purified, dephosphorylated, labeled with T4 polynucleotide kinase and use as dsDNA substrate. After labeling, dsDNA substrate was purified using Microspin S200 columns (Amersham). To obtain the ssDNA substrate, the labeled dsDNA was denatured by heating.

#### In Vitro Protein Binding Activity Assay

Binding assays were performed in 10 µl of a Mobility Shift Buffer (MSB) containing a final concentration of 10 mM Tris (pH 8.0), 25 mM KCl, 10 mM NaCl, 0.5 mM DTT, 10% glycerol, 100 µg/ml BSA. Approximately the same amount of each test molecule (10 ng) was used for each experiment; the amount of fusion proteins added is indicated in the figure legends. Binding was performed at 25°C for 5 min and then quickly cooled on ice. Binding reactions were then directly loaded onto a 4% polyacrylamide native gel. Electrophoresis was for two hours at 150 volts on a gel of 18 cm in length; running buffer was 1× TBE (0.1 M Tris, 83 mM H3BO3, 1 mM EDTA). Gels were dried and then subject to autoradiography. A small amount of bacterial RNase co-purify with the GST-HYL1 fusion protein and degrades the free (unprotected) RNA probes in Figure 2A.

### Nuclease Protection Assay

RNA was *in vitro* transcribed and radioactively trace-labeled (for quantification purposes) from linearized T7 promoter-containing vector using recombinant T7 RNA polymerase and [α-^32^P] ATP (GE Healthcare). Transcripts were gel-purified. 2 pmol of RNA was then dephosphorylated using calf intestinal phosphatase (New England Biolabs) according to manufacturer's protocol. Dephosphorylated RNAs were 5′-end labeled with T4 polynucleotide kinase (New England Biolabs) and [γ-^32^P] ATP (GE Healthcare). Gel-purified 5′-labeled RNAs were subsequently used for nuclease protection assays [41]. RNase V1 recognizes any 4-6-nt segment of polynucleotide backbone with an approximately helical conformation and cleaves leaving 5′-phosphates [43]. For the partial digest with RNase V1, 20 fmol (corresponding to 50,000 cpm) of RNA were centrifuged, washed, dried and resuspended in structure buffer (Ambion: 100 mM Tris at pH 7, 1 M KCl, 100 mM MgCl_2_). After annealing, 1 µL of tRNA (1 µg/µL, Ambion) was added, followed by the addition of increasing protein concentrations (50 nM, 150 nM and 500 nM). To ensure protein binding to RNA, samples were incubated for 15 min at room temperature. Then, 0.005 units of RNase V1 (Ambion) were added and the reactions were incubated for additional 10 min at room temperature. Reactions were stopped by ethanol/salt precipitation. Samples were loaded together with alkaline hydrolysis ladder and denaturing RNase A (Ambion) and RNase T1 (Boehringer Mannheim) digests of RNAs on denaturing RNA gels.

### RNA Isolation and Hybridizations

Total RNA was extracted using inflorescences (stages 1–12), as described elsewhere [28]. Northen blot analyses of mRNA accumulation were performed as described previously [51]. For the detection of small RNAs, 15 µg of total RNA samples were heat-treated in 1.5 volume of standard formamide buffer and loaded on 15% polyacrylamide (19∶1 acrylamide:bis-acrylamide) - 8 M urea - 0.5× TBE gel and separated by electrophoresis. The samples were electroblotted to hybond-NX membranes (GE healthcare) and fixed following a carbodiimide-mediated cross-linking procedure [52]. Pre-hybridization and hybridization was carried out in 5× SSC, 20 mM Na_2_HPO_4_ pH 7.2, 7% SDS, 2× Denhardt solution, 50 mg/ml denaturated hering DNA at 50°C. Filters were washed twice with 3X SSC, 25 mM NaH_2_PO_4_ pH 7.5, 5% SDS at 50°C for 10 min, followed by one to two washes with 1× SSC, 1% SDS at 50°C. Signals were visualized using a phosphorimager (Molecular Imager FX; Bio-Rad) for quantification. The random-primed ^32^P-labelled probes used for the detection of the ARF3, ARF4 and SCL6-III mRNAs have been described previously [48],[53]. For mi/tasiRNAs detection, DNA oligonucleotides whose sequences are complementary to individual mi/tasiRNAs were ^32^P-labeled with T4 polynucleotide kinase (New England Biolabs). For U6 detection the following oligonucleotide was used: 5′-AGGGGCCATGCTAATCTTCTC-3′.

## Supporting Information

Figure S1 Protection from RNase V1 digestion of SINE SB2 RNA by different dsRBPs. Prior to RNase V1 digestion, *in vitro* transcript of SB2 was subjected to protection by increasing concentrations of following dsRBPs: Dr2, HYL1, combination of Dr2 and HYL1, or DRB4. Regions protected by HYL1 are marked alongside short run gel (A), long run gel (B) and predicted folding pattern (C) by green bars. Three independent experiments gave similar results as the one presented. Nucleotides marked with asterisks seem to adopt more prominent helical structure upon protein binding and, therefore, become more prone to RNase V1 cleavage. (HL) represents a partially hydrolyzed RNA ladder. Denaturating RNase A and T1 digests give the position of pyrimidine and G residues respectively. The control lane shows untreated RNA samples and the (0) lane represent RNase V1 digestion without recombinant proteins added. A labeled 23-mer oligoribonucleotide was also loaded on the gel to help in band size determination. The binding of HYL1 to SB2 is weaker compared to SB1 or 11Dr2(7). In this case, both Dr2 and DRB4 are showing no effect on RNase V1 cleavage, suggesting their low *in vitro* binding affinity to SB2 RNA.
(4.82 MB TIF)

Figure S2 mRNA levels of HYL1, DCL1 and HEN1 genes involved in miRNA production are unchanged in the Col-0-SB1(18) transgenic line. (A) HYL1 mRNAs accumulate to similar levels in wild-type and Col0-SB1.7(18) line as indicated by Northern blot analyses using total RNA from aerial tissues of 14 days-old plants. (B) Similar results were obtained for HYL1, DCL1 and HEN1 transcript levels by SemiQ-RTPCR using flower total RNA as samples and Actin2 (ACT2) and Ubiquitin1 (UBQ1) as internal references, suggesting that the inhibition of miRNA production in SB1.7-expressing lines is not attributable to misexpression of one of the main genes from the miRNA pathway. First strand cDNA synthesis was done by using 0.8 µg of flower total RNA following treatment with DNAse (DNA-free kit; Ambion). PCRs were run for 25 cycles with the Actin2 (ACT2), Ubiquitin1 (UBQ1) and HYL1-specific primers and for 29 cycles for DCL1 and HEN1. For DCL1 the primers are positioned from each side of the miR162 target site, allowing amplification of the full-length DCL1 transcript only. Sequences of the primers are available upon request.
(0.46 MB TIF)
