# Supplementary figures and images for: SINE RNA Induces Severe Developmental Defects in Arabidopsis thaliana and Interacts with HYL1 (DRB1), a Key Member of the DCL1 Complex
Source: PLoS Genet. 2008 Jun 13;4(6):e1000096. doi: 10.1371/journal.pgen.1000096 (PMC2408557; doi:10.1371/journal.pgen.1000096)

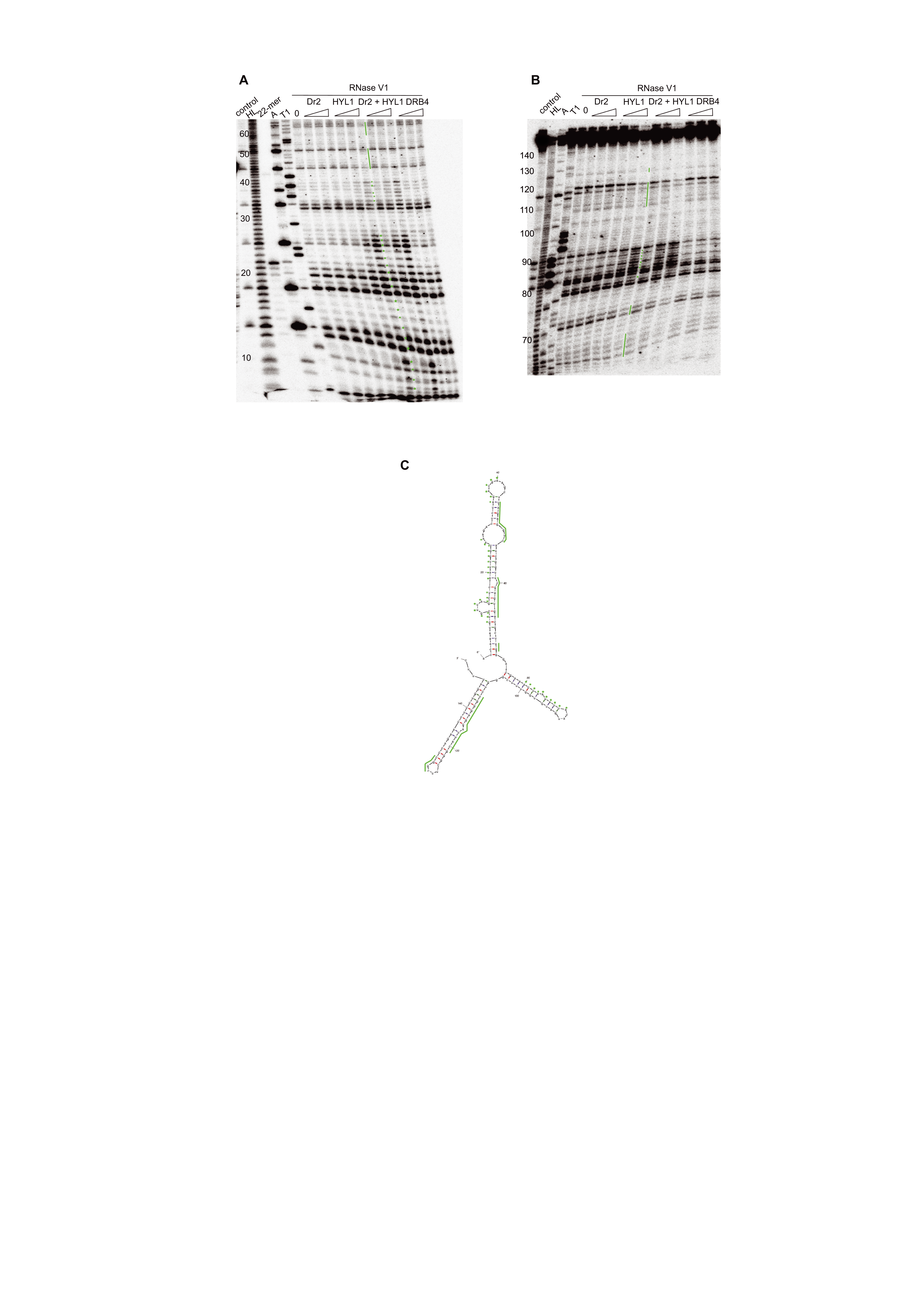

Supplement: Figure S1 — Protection from RNase V1 digestion of SINE SB2 RNA by different dsRBPs. Prior to RNase V1 digestion, in vitro transcript of SB2 was subjected to protection by increasing concentrations of following dsRBPs: Dr2, HYL1, combination of Dr2 and HYL1, or DRB4. Regions protected by HYL1 are marked alongside short run gel (A), long run gel (B) and predicted folding pattern (C) by green bars. Three independent experiments gave similar results as the one presented. Nucleotides marked with asterisks seem to adopt more prominent helical structure upon protein binding and, therefore, become more prone to RNase V1 cleavage. (HL) represents a partially hydrolyzed RNA ladder. Denaturating RNase A and T1 digests give the position of pyrimidine and G residues respectively. The control lane shows untreated RNA samples and the (0) lane represent RNase V1 digestion without recombinant proteins added. A labeled 23-mer oligoribonucleotide was also loaded on the gel to help in band size determination. The binding of HYL1 to SB2 is weaker compared to SB1 or 11Dr2(7). In this case, both Dr2 and DRB4 are showing no effect on RNase V1 cleavage, suggesting their low in vitro binding affinity to SB2 RNA. (4.82 MB TIF) [file pgen.1000096.s001.tif]

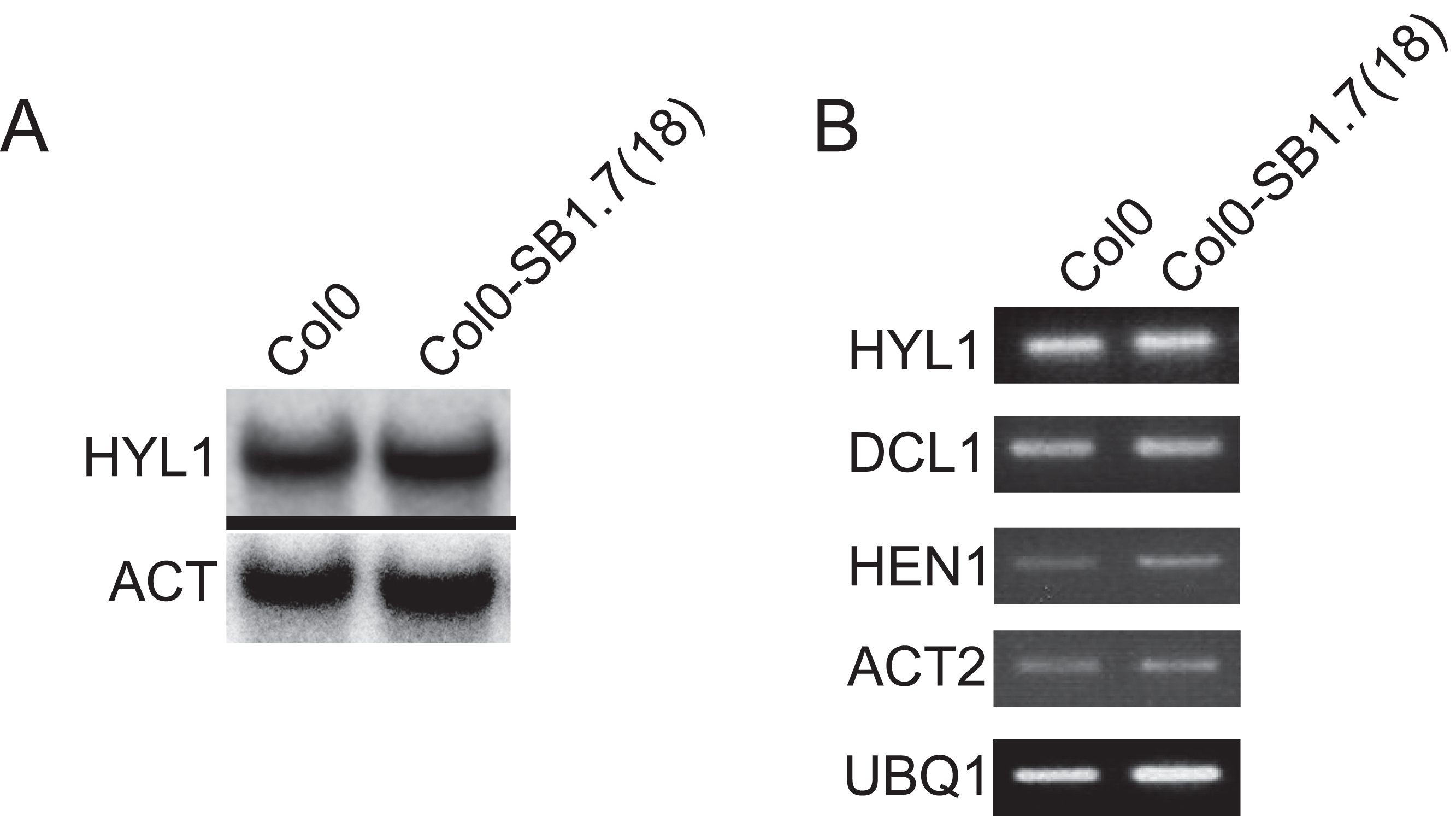

Supplement: Figure S2 — mRNA levels of HYL1, DCL1 and HEN1 genes involved in miRNA production are unchanged in the Col-0-SB1(18) transgenic line. (A) HYL1 mRNAs accumulate to similar levels in wild-type and Col0-SB1.7(18) line as indicated by Northern blot analyses using total RNA from aerial tissues of 14 days-old plants. (B) Similar results were obtained for HYL1, DCL1 and HEN1 transcript levels by SemiQ-RTPCR using flower total RNA as samples and Actin2 (ACT2) and Ubiquitin1 (UBQ1) as internal references, suggesting that the inhibition of miRNA production in SB1.7-expressing lines is not attributable to misexpression of one of the main genes from the miRNA pathway. First strand cDNA synthesis was done by using 0.8 µg of flower total RNA following treatment with DNAse (DNA-free kit; Ambion). PCRs were run for 25 cycles with the Actin2 (ACT2), Ubiquitin1 (UBQ1) and HYL1-specific primers and for 29 cycles for DCL1 and HEN1. For DCL1 the primers are positioned from each side of the miR162 target site, allowing amplification of the full-length DCL1 transcript only. Sequences of the primers are available upon request. (0.46 MB TIF) [file pgen.1000096.s002.tif]
